# Genome-wide SNP analysis identifies population structure and genetic differentiation in *Anopheles stephensi*

**DOI:** 10.1007/s00436-026-08745-8

**Published:** 2026-08-06

**Authors:** Melveettil Kishor Sumitha, Mariapillai Kalimuthu, Ashwani Kumar, Manju Rahi, Bhavna Gupta

**Affiliations:** 1 ICMR-NIVCR, Field Station, 4, Sarojini Street, Chinna Chokkikulam, Madurai, 625002 Tamil Nadu India; 2 ICMR-NIVCR, Medical Complex, Indira Nagar, Puducherry, 605006 India

**Keywords:** *Anopheles stephensi*, India, Genome-wide SNP, Genetic structure, Variant filtering

## Abstract

*Anopheles stephensi*, a major urban malaria vector in South Asia and the Arabian Peninsula, has recently spread to several African countries, raising significant public health concerns. Understanding its genetic structure is important for tracing invasion pathways; however, population genetic studies have been limited by sparse genome-wide datasets particularly from India, its native range. Here, we sequenced five *An. stephensi* samples from southern India and integrated them with 33 publicly available genomic datasets from India and Ethiopia to enable a comparison across native and invasive populations. Genome-wide analysis across 38 samples, revealed higher genetic diversity and evidence of broad-scale population differentiation among Indian and Ethiopian samples. Genetic differentiation between regions was moderate (FST = 0.14). A total of 813 SNPs were fixed (FST = 1) between populations, with 586 fixed in Ethiopian samples and 227 in Indian samples. Several fixed SNPs were concentrated in genes involved in sensory perception, ion transport, development, and transcriptional regulation, highlighting candidate genomic regions associated with population-specific differentiation that warrant further investigation. Overall, the results provide evidence of genetic differentiation between native and invasive populations and highlight genomic regions that may contribute to population-specific divergence in *An. stephensi*.

**Supplementary Information:**

The online version contains supplementary material available at https://doi.org/10.1007/s00436-026-08745-8.

## Introduction

*Anopheles stephensi* is a major urban malaria vector that breeds in small artificial freshwater habitats such as overhead tanks, sumps, barrels, and construction-site water pools. Historically, it has been prominent in India, the Middle East, and South Asia (Sharma et al. 1993; WHO 2007; Sinka et al. 2011). Since its first detection in Africa in Djibouti in 2012, *An. stephensi* has spread to Ethiopia (Carter et al. 2018), Sudan (Ahmed et al. 2021), Somalia (Ali et al. 2022), Kenya (Ochomo et al. 2023), Eritrea (Mhreteab et al. 2024) and Ghana (Afrane et al. 2023), raising concerns about urban malaria transmission. Understanding the origin and spread of invasive populations is important for vector control.

Comparative population genomics of other invasive mosquito vectors, such as *Aedes aegypti* and *Aedes albopictus* have significantly contributed in mapping invasion routes and migration histories (Bracco et al. 2007; Gloria-Soria et al. 2016; Vavassori et al. 2022). Applying similar genomic approaches to *An. stephensi* can clarify its origins and pattern of spread, thereby informing targeted control strategies. Although genome-wide datasets for *An. stephensi* are available (Samake et al. 2023; Dennis et al. 2025), those from India remain limited. Previous studies have generated genome-wide data from Indian populations (Thakare et al. 2022), and more recently population genomic analyses have identified extensive genomic variation, including structural variants and gene duplications that may contribute to local adaptation and population differentiation (Samano et al. 2025). Expanding genomic sampling from the native range remains essential for improving representation of genetic diversity and understanding evolutionary processes shaping populations.

In this study, we sequenced five *An. stephensi* samples from southern India [Accession ID PRJNA1320674 (SAMN51163023, SAMN51163024, SAMN51163025, SAMN51163026, SAMN51163027)] and integrated with 33 publicly available datasets (India, *n* = 9; Ethiopia, *n* = 23) and one laboratory strain from Pakistan (SDA500). We aimed to characterize the population structure and genetic differentiation of *An. stephensi* across native (India) and invasive (Ethiopia) populations using genome-wide SNP data and to identify genomic regions associated with population-specific divergence. Importantly, the newly generated genomes originate from southern Indian locations (Madurai and Thiruvananthapuram) that are not represented in previously published datasets, which primarily include samples from Chennai, Bengaluru, and Mangalore (BioProject ID: PRJNA746765). This expanded geographic coverage improves representation of the native range, enables evaluation of previously reported population patterns across a broader spatial scale, and contributes additional genomic data from southern India.

## Materials and methods

Five *An. stephensi* individuals were collected from southern India (Supplementary Table 1), with each genome representing a single mosquito. Genomic DNA was extracted using the DNeasy Blood and Tissue Kit (Qiagen), quantified with a Qubit fluorometer, and used to prepare libraries with the QIASeq FX DNA Library Kit (Qiagen). Libraries were adapter-ligated, amplified with six cycles of indexing PCR, purified using CamSelect NGS beads, and quality-checked on an Agilent TapeStation 4150. Sequencing was performed on an Illumina NovaSeq 6000 platform (150 bp paired-end). Raw reads from these samples, along with 33 publicly available datasets were processed (Supplementary Table 1) using a standardized pipeline. The raw reads were quality-filtered with FastP v0.20.1 (Chen et al. 2018) using a minimum length of 36 bp, Phred quality score ≥ 20, which were considered high-quality. Quality of reads was assessed with FastQC v0.11.9 (Andrews 2010), and the high-quality reads were aligned to the *An. stephensi* reference genome (GCF_013141755.1) (Chakraborty et al. 2021) using BWA-MEM (v0.7.17) (Li 2017). Duplicates were removed with Picard v3.3.0 (https://broadinstitute.github.io/picard/), and variants were called using GATK HaplotypeCaller (McKenna et al. 2010) in GVCF mode, followed by joint genotyping. Variant annotation was performed using SnpEff v4.3t (Cingolani et al. 2012). Only autosomal biallelic SNPs with < 10% missing data and a minor allele frequency > 0.05 were retained for further analyses.

Population genomic analyses were performed using a linkage disequilibrium–pruned SNP dataset generated in PLINK v1.9 (50-kb sliding window, 5-SNP step, r² < 0.2) (Purcell et al. 2007). The pruned dataset was used for principal component analysis (PCA) in PLINK and for population structure inference using fastSTRUCTURE (Raj et al. 2014), with K values ranging from 1 to 5 and 20 replicates per K. Genetic differentiation was quantified by estimating Weir and Cockerham’s *F*_ST_ (Weir and Cockerham 1984) for each SNP. Genes containing fixed SNPs (*F*_ST_ = 1) were annotated and functionally enriched using the DAVID functional annotation tool (Dennis et al. 2003; Sherman et al. 2022).

## Results and discussion

A total of ~ 5.07 million high-quality SNPs with < 10% missing genotypes and a minor allele frequency > 0.05 were retained for population genomic analyses. The overall genotyping rate was 0.97, indicating approximately 3% missing data per sample. The transition/transversion (Ts/Tv) ratio was 1.26, consistent with values reported for other mosquito species (Wondji et al. 2007; Acharya et al. 2024).

Principal component analysis suggested four distinct genetic clusters (Fig. 1), including two clusters among Indian samples (*n* = 14), a single Ethiopian cluster (*n* = 23), and the reference strain SDA500 positioned intermediate between Ethiopian and one Indian cluster (Fig. 1). This pattern was further supported by fastSTRUCTURE analysis, which identified K = 4 as the best-supported model (Fig. 2a, b). The presence of two genetically distinct Indian clusters is consistent with the higher nucleotide diversity and pronounced sub-structure observed within Indian populations, reflecting the native range of *An. stephensi*. High genetic diversity among *An. stephensi* populations has been reported previously in India and is thought to be shaped by geographic separation, ecological heterogeneity, and demographic history across urban and semi-arid environments (Sharma et al. 2016; Thakare et al. 2022). Although the inclusion of samples from previously unsampled locations expands the geographic representation of the native range, the limited number of specimens per location may not fully capture local genetic diversity or fine-scale population structure. Future studies incorporating larger sample sizes and broader geographic coverage will be important for resolving population structure across the native range of *An. stephensi*.

**Fig. 1:**
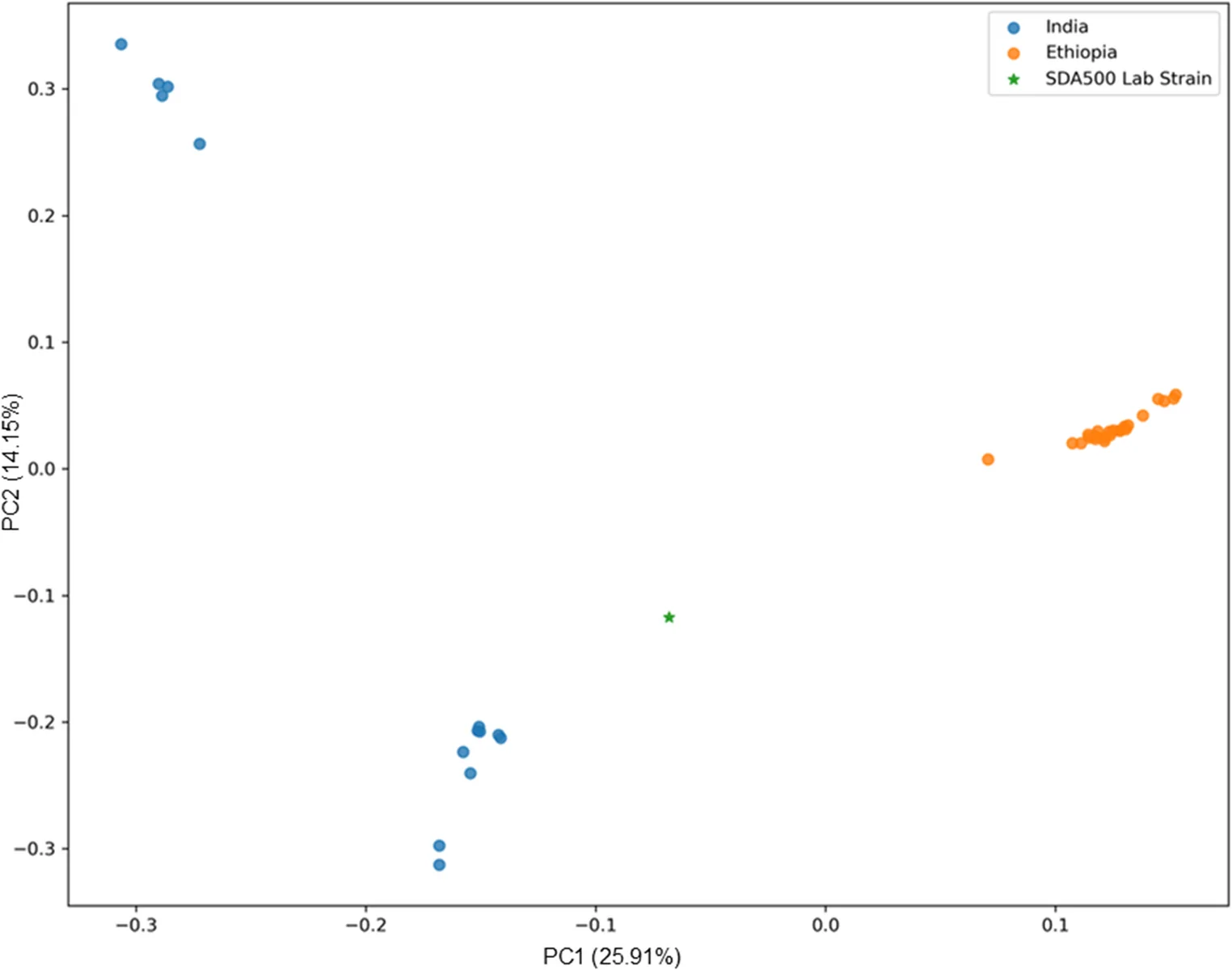
Principal component analysis of SNP variation among *Anopheles stephensi* samples

**Fig. 2:**
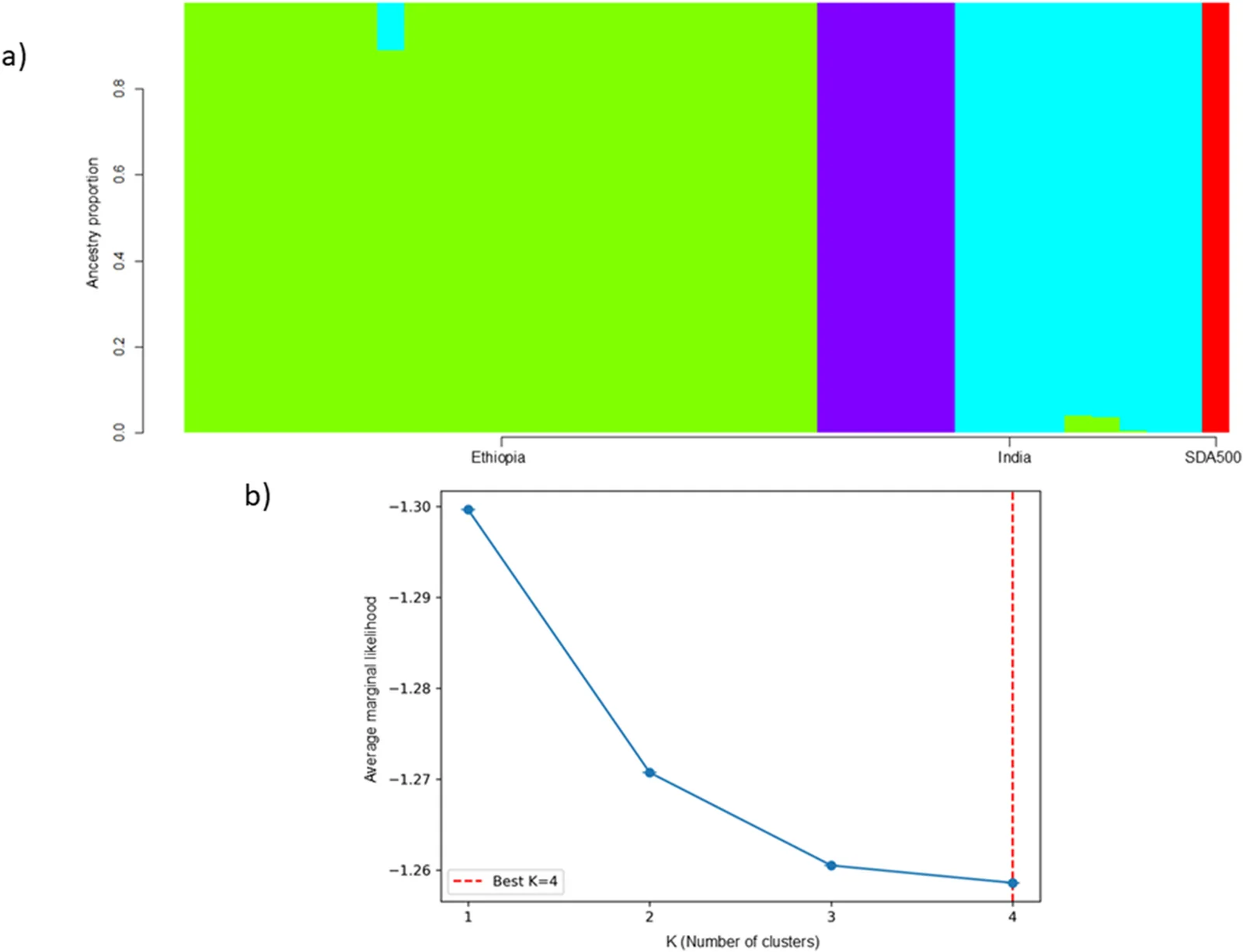
Population genetic structure of *Anopheles stephensi* inferred using fastSTRUCTURE based on genome-wide SNP data. (a) Ancestry proportions at K = 4, and (b) plot showing marginal likelihood values for K ranging from 1 to 4 used to determine the optimal number of clusters

Ethiopian samples formed a relatively homogeneous cluster with reduced genetic diversity and showed moderate but significant differentiation from Indian populations (*F*_ST_ = 0.14). This pattern is characteristic of recently introduced populations and is likely driven by founder effects and limited geographic sampling, as documented in earlier studies (Surendran et al. 2018). However, environmental differences between native and invaded ranges may also contribute to the observed differentiation, although further studies are required to evaluate this possibility.

The laboratory strain SDA500 occupied an intermediate position between Ethiopian and one Indian cluster in both PCA and fastSTRUCTURE analyses. However, this pattern should be interpreted cautiously because prolonged laboratory colonization may have altered allele frequencies through genetic drift, adaptation to laboratory conditions, and reduced effective population size. Therefore, SDA500 was included primarily as a reference genome resource from South Asia rather than as a field sample from Pakistan. While the observed clustering provides additional context for interpreting regional genetic relationships, definitive reconstruction of invasion routes will require broader sampling of contemporary field populations across South Asia, the Middle East, and Africa.

Despite moderate overall differentiation, ~ 0.18 million SNPs exhibited *F*_ST_ > 0.5 (Fig. S1), indicating loci with strong divergence. Of these, 813 SNPs were fixed in a population-specific manner (*F*_ST_ = 1), with 586 fixed in Ethiopian samples and 227 fixed in Indian samples. These variants mapped to 312 genes. None of the fixed SNPs were predicted to have high-impact effects; most were synonymous or located in non-coding regions. Notably, fixed SNPs were unevenly distributed, with 14 genes harboring more than ten fixed SNPs each (Table 1), suggesting localized genomic regions of population-specific divergence. Ancestral versus derived states of fixed SNPs were not determined in this study due to the absence of an appropriate outgroup; therefore, interpretations are limited to patterns of population-specific fixation.

**Table 1:** Details of genes harbouring more than 10 population-specific fixed SNPs

Gene ID	Gene name/annotation	No. of fixed SNPs	Population fixed	Variant types (summary)	Highest impact
118,513,631	Uncharacterized	29	Both	Upstream gene variant, intron variant	Modifier
118,506,036	Krueppel-like factor luna	28	Both	Upstream gene variant, intron variant	Modifier
118,509,863	Uncharacterized	20	Both	Intron variant	Modifier
118,511,552	Dystroglycan	19	Ethiopia	Intron variant	Modifier
118,504,075	sodium-dependent nutrient amino acid transporter 1-like	17	Ethiopia	Upstream gene variant	Modifier
118,513,649	potassium voltage-gated channel protein Shaw-like	12	Both	Upstream gene variant, intron variant, downstream gene variant, 3 prime UTR variant	Modifier
118,504,283	FAT atypical cadherin kugelei	11	Ethiopia	Intron variant	Modifier
118,514,333	semaphorin-2 A-like	10	Ethiopia	Intron variant	Modifier
Intergenic Variants (≥ 10)					
LOC118509564-LOC118509566	polycomb group protein Psc-like	14			
LOC118512732-LOC118513288	general odorant-binding protein 45-like Uncharacterized	13			
LOC118509034-LOC118511858	CUGBP Elav-like family member bruno 3 odorant receptor 82a-like	11			

Several of these genes are associated with sensory perception, including odorant receptors and odorant-binding proteins, which may influence host-seeking behavior and habitat selection (Table 1). Additional genes were linked to ion channels and nutrient transporters, such as potassium voltage-gated channel Shaw-like and sodium-dependent nutrient amino acid transporter 1-like, which may affect neuronal signaling and metabolic processes. Dystroglycan, FAT atypical cadherin kugelei, and semaphorin-2 A-like, have previously been implicated in developmental and structural processes (Deng et al. 2003; Schneider et al. 2006; Bennett and Harvey 2006; Bates and Whitington 2007; Viktorinová et al. 2009) and therefore represent candidate genes associated with population differentiation. Fixed variants were also identified in transcriptional regulators, such as Krueppel-like factor luna and polycomb group protein Psc-like, which could contribute to broader differences in gene regulation between populations.

Importantly, patterns observed in PCA were concordant with the distribution of fixed SNPs. Several highly differentiated loci were located within or near genes associated with sensory perception, ion transport, and regulatory processes. Similar enrichment of chemosensory and environmental response genes has been reported in other invasive mosquito species undergoing rapid range expansion (Lombardo et al. 2017; Gomulski et al. 2020; Lozada-Chávez et al. 2025; Menon et al. 2025). While the present study focused on SNP variation, recent population genomic analyses of *An. stephensi* have demonstrated that structural variants, including gene duplications and other large-scale genomic rearrangements, contribute substantially to genomic diversity and may play important roles in adaptation (Samano et al. 2025). Consequently, some highly differentiated or fixed SNPs identified in the present study may be linked to nearby structural variants that are under selection. Future studies integrating SNP, structural variant, and copy-number variation analyses across native and invasive populations will provide a more comprehensive understanding of the genomic mechanisms underlying population differentiation and adaptation in *An. stephensi*.

Although inferences are constrained by the limited number of newly sequenced genomes, their inclusion provides an independent dataset that complements previously available data. The broad patterns observed in the present study are generally consistent with previous population genomic analyses (Acford-Palmer et al. 2023), indicating that these patterns are robust across datasets generated from different geographic locations and sequencing efforts. The newly sequenced specimens were included to expand geographic representation of the native range rather than to estimate within-population genetic diversity; accordingly, interpretations are focused on broad-scale patterns of differentiation among the sampled populations. Future studies incorporating larger sample sizes and broader geographic coverage across native and invaded ranges will be important to further refine these inferences and support genomics-informed vector surveillance and control strategies.

## Supplementary Information

Below is the link to the electronic supplementary material.

Supplementary Material 1 (TXT 3.81 KB)

Supplementary Material 2 (TIF 309 KB)

Supplementary Material 3 (XLSX 9.36 KB)

## Data Availability

The dataset(s) supporting the conclusions of this article is(are) included within the article (and its additional file(s)).

## References

[CR1] Acford-Palmer H, Phelan JE, Tadesse FG et al (2023) Identification of two insecticide resistance markers in Ethiopian *Anopheles stephensi* mosquitoes using a multiplex amplicon sequencing assay. Sci Rep 13:5612. 10.1038/s41598-023-32336-7 PMC10076309 37019918

[CR2] Acharya P, Singh US, Rajamannar V et al (2024) Genome resequencing and genome-wide polymorphisms in mosquito vectors *Aedes aegypti* and *Aedes albopictus* from south India. Sci Rep 14. 10.1038/s41598-024-71484-2 PMC11447132 39358370

[CR3] Afrane YA, Abdulai A, Mohammed AR et al (2023) First detection of *Anopheles stephensi* in Ghana using molecular surveillance. bioRxiv. 10.1101/2023.12.01.569589

[CR4] Ahmed A, Khogali R, Elnour M-AB et al (2021) Emergence of the invasive malaria vector *Anopheles stephensi*, in Khartoum State, Central Sudan. Parasites Vectors 14:511. 10.1186/s13071-021-05026-4 PMC8487507 34600584

[CR5] Ali S, Samake JN, Spear J, Carter TE (2022) Morphological identification and genetic characterization of *Anopheles stephensi* in Somaliland. Parasites Vectors 15:247. 10.1186/s13071-022-05339-y PMC9270831 35804441

[CR6] Andrews S (2010) FastQC: a quality control tool for high throughput sequence data. https://www.bioinformatics.babraham.ac.uk/projects/fastqc/

[CR7] Bates KE, Whitington PM (2007) Semaphorin 2a secreted by oenocytes signals through plexin B and plexin A to guide sensory axons in the *Drosophila* embryo. Dev Biol 302:522–535. 10.1016/j.ydbio.2006.10.015 17109838

[CR8] Bennett FC, Harvey KF (2006) Fat cadherin modulates organ size in *Drosophila* via the Salvador/Warts/Hippo signaling pathway. Curr Biol 16:2101–2110. 10.1016/j.cub.2006.09.045 17045801

[CR9] Bracco JE, Capurro ML, Lourenço-de-Oliveira R, Sallum MAM (2007) Genetic variability of *Aedes aegypti* in the Americas using a mitochondrial gene: evidence of multiple introductions. Mem Inst Oswaldo Cruz 102:573–580. 10.1590/s0074-02762007005000062 17710301

[CR10] Carter TE, Yared S, Gebresilassie A et al (2018) First detection of *Anopheles stephensi* Liston, 1901 (Diptera: Culicidae) in Ethiopia using molecular and morphological approaches. Acta Trop 188:180–186. 10.1016/j.actatropica.2018.09.001 30189199

[CR11] Chakraborty M, Ramaiah A, Adolfi A et al (2021) Hidden genomic features of an invasive malaria vector, *Anopheles stephensi*, revealed by a chromosome-level genome assembly. BMC Biol 19:28. 10.1186/s12915-021-00963-z PMC7876825 33568145

[CR12] Chen S, Zhou Y, Chen Y, Gu J (2018) fastp: an ultra-fast all-in-one FASTQ preprocessor. Bioinformatics 34:i884–i890. 10.1093/bioinformatics/bty560 PMC6129281 30423086

[CR13] Cingolani P, Platts A, Wang LL et al (2012) A program for annotating and predicting the effects of single nucleotide polymorphisms, SnpEff: SNPs in the genome of *Drosophila melanogaster* strain w^1118^; iso-2; iso-3. Fly 6:80–92. 10.4161/fly.19695 PMC3679285 22728672

[CR14] Deng W-M, Schneider M, Frock R et al (2003) Dystroglycan is required for polarizing the epithelial cells and the oocyte in *Drosophila*. Development 130:173–184. 10.1242/dev.00199 12441301

[CR15] Dennis G, Sherman BT, Hosack DA et al (2003) DAVID: Database for Annotation, Visualization, and Integrated Discovery. Genome Biol 4:P3 PMC193660 12734009

[CR16] Dennis TPW, Sulieman JE, Abdin M et al (2025) The origin, invasion history and resistance architecture of *A**nopheles stephensi* in Africa. bioRxiv. 10.1101/2025.03.24.644828

[CR17] Gloria-Soria A, Ayala D, Bheecarry A et al (2016) Global genetic diversity of *Aedes aegypti*. Mol Ecol 25:5377–5395. 10.1111/mec.13866 PMC5123671 27671732

[CR18] Gomulski LM, Manni M, Carraretto D et al (2020) Transcriptional variation of sensory-related genes in natural populations of *Aedes albopictus*. BMC Genomics 21:547. 10.1186/s12864-020-06956-6 PMC7430840 32767966

[CR19] Li H (2017) BWA-MEM v0.7.17

[CR20] Lombardo F, Salvemini M, Fiorillo C et al (2017) Deciphering the olfactory repertoire of the tiger mosquito *Aedes albopictus*. BMC Genomics 18:770. 10.1186/s12864-017-4144-1 PMC5637092 29020917

[CR21] Lozada-Chávez AN, Lozada-Chávez I, Alfano N et al (2025) Adaptive genomic signatures of globally invasive populations of the yellow fever mosquito *Aedes aegypti*. Nat Ecol Evol 9:652–671. 10.1038/s41559-025-02643-5 PMC11976285 40155778

[CR22] McKenna A, Hanna M, Banks E et al (2010) The Genome Analysis Toolkit: A MapReduce framework for analyzing next-generation DNA sequencing data. Genome Res 20:1297–1303. 10.1101/gr.107524.110 PMC2928508 20644199

[CR23] Menon R, Ignell R, Hill SR (2025) Differential expression of antennal chemosensory genes related to host preference of *Culex pipiens* biotypes. Parasit Vectors 18:372. 10.1186/s13071-025-07028-y PMC12459065 40988078

[CR24] Mhreteab A, Yenus M, Mebrahtu A (2024) First detection of *Anopheles stephensi* (Diptera: Culicidae) in Gash Barka Region: Eritrea. Int J Biol Sci 6:176–182. 10.33545/26649926.2024.v6.i1c.203

[CR25] Ochomo EO, Milanoi S, Abong’o B et al (2023) Detection of *Anopheles stephensi* mosquitoes by molecular surveillance, Kenya. Emerg Infect Dis 29. 10.3201/eid2912.230637 PMC10683825 37966106

[CR26] Purcell S, Neale B, Todd-Brown K et al (2007) PLINK: A tool set for whole-genome association and population-based linkage analyses. Am J Hum Genet 81:559–575. 10.1086/519795 PMC1950838 17701901

[CR27] Raj A, Stephens M, Pritchard JK (2014) fastSTRUCTURE: Variational inference of population structure in large SNP data sets. Genetics 197:573–589. 10.1534/genetics.114.164350 PMC4063916 24700103

[CR28] Samake JN, Lavretsky P, Gunarathna I et al (2023) Population genomic analyses reveal population structure and major hubs of invasive *Anopheles stephensi* in the Horn of Africa. Mol Ecol 32:5695–5708. 10.1111/mec.17136 PMC12822364 37795951

[CR29] Samano A, Kumar N, Liao Y et al (2025) Genome structural variants shape adaptive success of an invasive urban malaria vector *Anopheles stephensi*. Mol Biol Evol 42:msaf140. 10.1093/molbev/msaf140 PMC12198770 40476664

[CR30] Schneider M, Khalil AA, Poulton J et al (2006) Perlecan and Dystroglycan act at the basal side of the *Drosophila* follicular epithelium to maintain epithelial organization. Development 133:3805–3815. 10.1242/dev.02549 PMC2753471 16943280

[CR32] Sharma SN, Subbarao SK, Choudhury DS, Pandey KC (1993) Role of *An. culicifacies* and *An. stephensi* in malaria transmission in urban Delhi. Indian J Malariol 30:155–168 8131883

[CR31] Sharma R, Sharma A, Kumar A et al (2016) Population genetic structure of urban malaria vector *Anopheles stephensi* in India. Infect Genet Evol 39:35–44. 10.1016/j.meegid.2016.01.003 26777030

[CR33] Sherman BT, Hao M, Qiu J et al (2022) DAVID: a web server for functional enrichment analysis and functional annotation of gene lists (2021 update). Nucleic Acids Res 50:W216–W221. 10.1093/nar/gkac194 PMC9252805 35325185

[CR34] Sinka ME, Bangs MJ, Manguin S et al (2011) The dominant *Anopheles* vectors of human malaria in the Asia-Pacific region: occurrence data, distribution maps and bionomic précis. Parasites Vectors 4:89. 10.1186/1756-3305-4-89 PMC3127851 21612587

[CR35] Surendran SN, Sivabalakrishnan K, Gajapathy K et al (2018) Genotype and biotype of invasive *Anopheles stephensi* in Mannar Island of Sri Lanka. Parasit Vectors 11:3. 10.1186/s13071-017-2601-y PMC5753456 29298698

[CR36] Thakare A, Ghosh C, Alalamath T et al (2022) The genome trilogy of *Anopheles stephensi*, an urban malaria vector, reveals structure of a locus associated with adaptation to environmental heterogeneity. Sci Rep 12:3610. 10.1038/s41598-022-07462-3 PMC8897464 35246568

[CR37] Vavassori L, Honnen A, Saarman N et al (2022) Multiple introductions and overwintering shape the progressive invasion of *Aedes albopictus* beyond the Alps. Ecol Evol 12. 10.1002/ece3.9138 PMC9313497 35903757

[CR38] Viktorinová I, König T, Schlichting K, Dahmann C (2009) The cadherin Fat2 is required for planar cell polarity in the *Drosophila* ovary. Development 136:4123–4132. 10.1242/dev.039099 19906848

[CR39] Weir BS, Cockerham CC (1984) Estimating F-statistics for the analysis of population structure. Evolution 38:1358. 10.2307/2408641 28563791

[CR40] WHO (2007) Anopheline species complexes in South and South-East Asia. SEARO. World Health Organization Regional Office for South-East Asia, New Delhi

[CR41] Wondji CS, Hemingway J, Ranson H (2007) Identification and analysis of single nucleotide polymorphisms (SNPs) in the mosquito *Anopheles funestus*, malaria vector. BMC Genomics 8. 10.1186/1471-2164-8-5 PMC1781065 17204152

